# Three-dimensional microscopic comparison of wear behavior between immature and mature enamel: an in vitro study

**DOI:** 10.1186/s12903-023-02751-3

**Published:** 2023-01-25

**Authors:** Kanae Wada, Manhal Ijbara, Nesreen A. Salim, Junichiro Wada, Tsutomu Iwamoto

**Affiliations:** 1 Department of Pediatric Dentistry/Special Needs Dentistry, Tokyo Medical and Dental University (TMDU), 1-5-45 Yushima, Bunkyo-ku, Tokyo, Japan; 2 Hail Dental Center and King Khalid Hospital, Ministry of Health, 55425 2910 Hail, Kingdom of Saudi Arabia; 3 Prosthodontic Department, Faculty of Dentistry, School of Dentistry, The University of Jordan Hospital, The University of Jordan, Amman, Jordan; 4 Removable Partial Denture Section, Graduate School, Tokyo Medical and Dental University (TMDU), 1-5-45 Yushima, Bunkyo-ku, Tokyo, 113-8549 Japan

**Keywords:** Tooth wear, Dental enamel, Permanent dentition, Adolescents, Microscopy

## Abstract

**Background:**

Dental enamel, the hardest outermost layer of a human tooth, is subjected to occlusal forces throughout life during different oral function as talking, mastication etc. Due to this continuous stress, wear causes the loss of this protective shell. This study aimed to detect microscopic differences in enamel’s wear behavior among different age groups of adolescents and adults.

**Aims and methods:**

Enamel specimens from immature open-apex and mature closed-apex premolars were subjected to simulated occlusal wear of impact and sliding wear test ISWT. Upper and lower enamel specimens were made to come in contact under controlled conditions. The enamel specimens’ surfaces were examined using different microscopes. The upper and lower specimens were subjected to the following tests; pre-test light microscopy examination, enamel specimens’ preparation for ISWT, scanning laser confocal microscopy of upper specimens, three-dimensional (3D) colored laser microscope and a Profilometer imaging of the lower specimens.

**Results:**

Wear characteristics, including wear areas, crater depths, and relation to enamel microstructures, differed among different age groups. Immature enamel from the upper specimens was more resistant to chipping than mature enamel with no statistically significant wear area difference. The immature enamel craters from the lower specimens were wider and deeper than those in the mature enamel; the wear areas in the mature enamel in the lower specimens were almost flat and smooth. The wear areas in the immature enamel in the lower specimens were significantly larger than those in the mature enamel.

**Conclusions:**

Wear characteristics of the immature enamel are different from those of the mature enamel. Hence, it should be repaired using restorative materials with compatible wear properties.

## Background

Wear is a growing problem among adolescents, thus posing new clinical challenges for its treatment and prevention [1]. The process of tooth wear is complex, unpredictable, and multifactorial [2]. Other than caries and trauma, other causes of loss of hard tooth material include the deterioration of enamel and dentin on the occlusal surfaces of the teeth [2] Enamel loss can have detrimental effects on oral function and quality of life [3].

Many studies have investigated tooth wear in mature teeth [4, 5], however scientific literature of tooth wear in immature teeth in children and adolescents is scant [6]. Tooth wear is a growing problem in terms of clinical treatment and management in the young population [6]. Various factors that have been implicated as being etiologic and/or associated with the processes of dental wear in adolescents and adults include functional (i.e., Chewing) or parafunctional habits (eg, bruxism) and orthodontic patterns of mandibular movement, such as canine guidance, anterior guidance, or group function. Similarly, diet (ie, coarse and acidic substances as soft drinks), diseases (eg, gastric conditions, anorexia nervosa), salivary composition (ie, buffer capacity, secretion rate, and variations in calcium ion concentration), and occupational environment (eg, airborne abrasives, acid). Extensive wear is also associated with age and gender [3, 7]. Understanding the differences in characteristics between mature and immature teeth is essential for selecting appropriate materials for restoration that have superior friction matching qualities [8].

Due to the undergoing intraoral chemical changes and the complex hierarchical structure of the tooth, the enamel’s physical and chemical properties change over time in the oral cavity [9]. Changes in the chemical composition, known as “post-eruptive maturation,” influence the enamel’s chemical and physical properties [10, 11]. Teeth continue to mature during childhood, and by adulthood, the physical properties, such as tooth hardness and modulus of elasticity, are improved [10, 11].

In clinical practice, trauma and congenital malformations, including defects such as Molar Incisor Hypomineralization, sometimes require construction and repair after tooth eruption [12]. On the other hand, immature teeth have weak physical properties and may be markedly worn out by the antagonist’s teeth during function, where preventive and curative protocols are paramount using appropriate restorative materials [6].

Wear resistance and microcrack behavior are important physical properties [6]. In light of this, we proposed that the features of wear-induced microcracks in enamel exposed to the same wear-stimulating circumstances in the oral cavity will vary depending on the post-eruptive age. This study aimed to compare enamel wear characteristics in immature (teeth with open apex) and mature teeth (fully formed, closed-apex) utilizing a simulated wear test under controlled conditions followed by multiple microscopic imaging. The null hypothesis was “Enamel specimens derived from adolescents and adults if exposed to same wear simulating conditions will exhibit similar wear characteristics”.

## Materials and methods

The ethics committee of Tokyo Medical and Dental University (Institutional Research Board approval number 1032) approved this in vitro study. All study related procedures were carried out in accordance with the principles of Declaration of Helsinki. The authors of this paper declare no conflict of interests in association with the studies (Form no. 965-6). The human teeth specimens were extracted for reasons other than this study, mostly for orthodontic reasons, stored in a disinfectant solution in a tooth bank at TMDU. Patients/caregivers informed consent was obtained voluntarily at all times. Teeth were collected from children and adults who are bona fide TMDU dental patients and were treated for reasons other than this study. IRB number for this study deemed sufficient as the informed consents were obtained previously at time of extraction.

### Specimen preparation

The specimens were prepared in this study as described previously [4, 13]. Enamel specimens derived from 21 caries-free immature premolars (IM group; n = 21; open root apex) from individuals aged 9–15 years and 21 mature premolars (M group; n = 21; closed apex) from individuals aged 25–30 years were used in this study. The teeth had been extracted for orthodontic purposes and were stored in Hank’s balanced solution. The teeth crowns were examined by a light microscope to exclude any teeth with existing cracks. So, the inclusion criteria for all teeth are to be caries free and crack free with no signs of existing wear or fractures. The exclusion criterion was presence of caries. Incipient caries would affect the contact areas on mesial or distal surfaces or in the occlusal fissures. In this study, specimens’ preparation was performed on the buccal surface of cusps. None of the cervical, the proximal nor the occlusal areas were included.

The roots of each tooth were cut using a high-speed diamond fissure bur with sufficient water cooling. The pulp cavities were cleaned, etched, and filled with flowable resin composite (Metafill Flo, Sun Medical Co., Ltd., Tokyo, Japan).

**Fig. 1:**
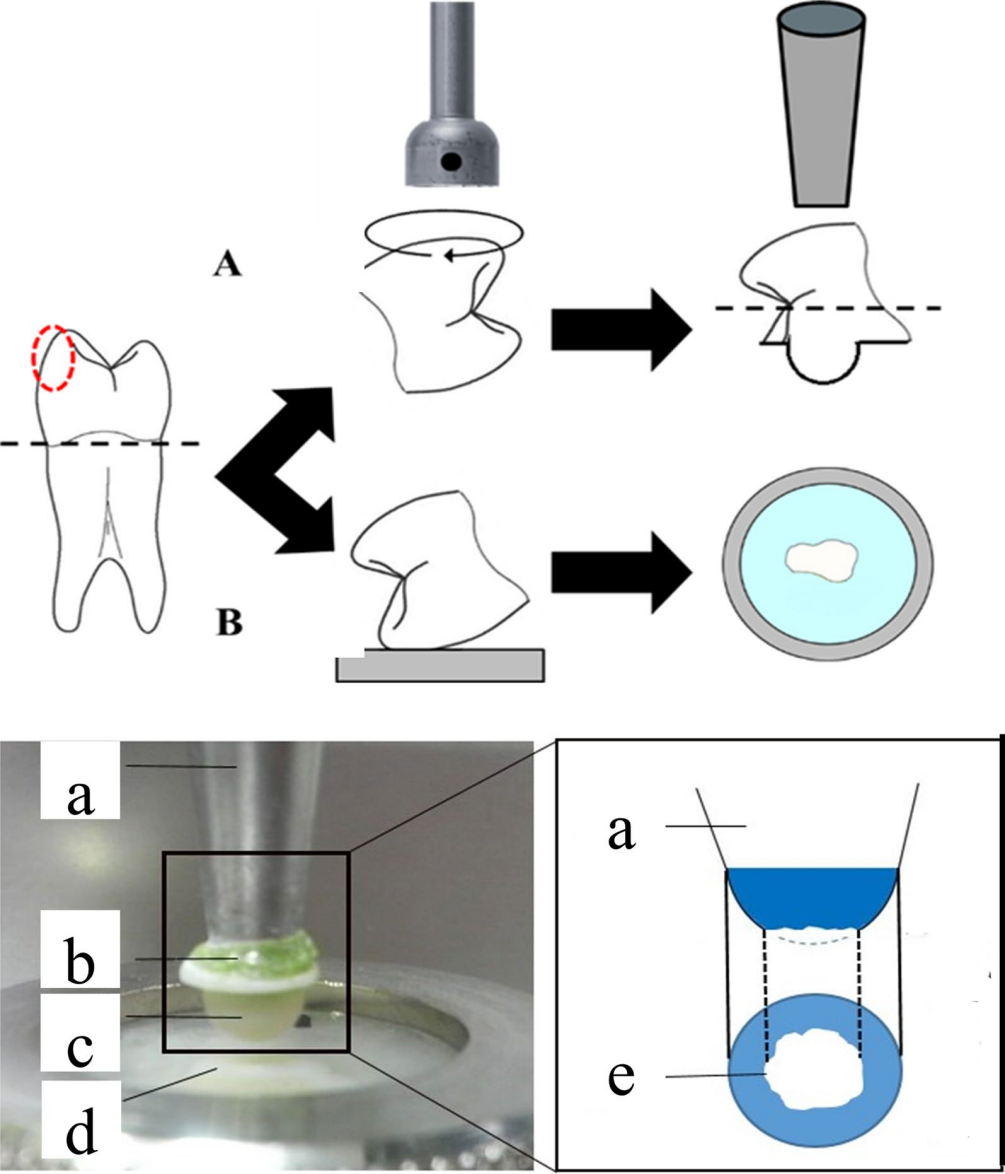
Sample preparation and settings for wear test; **a**: metal stylus, **b**: adhesive material, **c**: upper specimen: immature or mature enamel, **d**: lower specimen: immature or mature enamel, **e** top view of the worn enamel surface; wear area

The upper enamel specimens were prepared from the buccal halves of the premolar crowns. Each half was bonded to a metal stylus using Superbond C&B (Sun Medical Co., Ltd., Tokyo, Japan). Using a custom-made fine-grit cup-shaped diamond bur (Hinatawada Seimitsu MFG. Co., Ltd, Tokyo, Japan) under water cooling, the cusps’ bucco-occlusal surfaces were ground to a hemisphere up to 5 mm in diameter (Fig. 1).

The lower specimens of each group were prepared by embedding each crown in clear self-cure resin (Unifast III Clear, GC Corp, Tokyo, Japan). The crown was positioned in the center of an acrylic ring with the buccal surface placed against a glass slab surface (Fig. 1). After the resin was cured, the ring’s lower surface was polished using abrasive papers (grit order of: 600, 800, 1000, 1200, and 1500) along with copious irrigation to create an exposed enamel window. The bucco-occlusal cuspal area was exposed (minimum dimensions of the window: 2 mm × 2 mm) through the cured acrylic resin glossy surface. After the preparation, all the specimens were stored in Hank’s balanced solution before placement in the upper and lower compartments of a wear-simulating machine (Fig. 1). The hemi-spherically prepared upper enamel specimen represents a maxillary tooth cusp impacting and sliding against the lower specimen’s flattened enamel, which represents an opposing mandibular flat area in a simulated chewing cycle of the oral cavity.

### Impact sliding wear testing

Metal styli holding the upper specimens were placed on the power arms in the upper compartment of an impact sliding wear testing (ISWT) machine (ISWT K655-07, Tokyo Giken, Japan). The ISWT methodology was adapted from previous studies [4, 14]. The lower specimens were fixed at the bottom of a water-filled tank in the machine’s lower compartment. The machine could accept three specimens on each side. It was set to direct the upper specimens to drop onto the enamel window of the lower specimens from a height of 1 mm at a pressure of 30 N. The upper specimens will slide horizontally for 1 mm while in contact with the lower specimens before rising to begin a new cycle for a total of 20,000 cycles. The entire impacting sliding test took place below the water level in the machine tank, with the water temperature (37.5 Cº) and level checked regularly to prevent heating and to compensate evaporated water. The lower specimens were covered in a polymethyl methacrylate slurry (PMMA; MBX-50 Sekisui Plastics Co., Ltd., Tokyo, Japan) mixed with tap water (weight ratio, 1:1) to mimic a food bolus. In order to mimic mastication of the oral cavity in- vitro two movements were applied in the cycles. The vertical movement mimics the initial bite or cutting of food particles. On the other hand, horizontal movement mimics the grinding to soften food particles. These movements were unified to all specimens.

The following protocol was followed after every 5000 cycles: the upper specimens were removed, washed, and gently dried using absorbent tissues. An impression was made using a custom-made mini-tray with a light body wash polyvinyl impression material (Fusion II Wash Type, GC Corp). The lower specimens were kept in place and checked for the correct position. The slurry was washed off and renewed every 5000 cycles. The upper specimens were placed back in the machine to continue the wear cycling for 20,000 cycles.

### Evaluation

#### Upper specimens

**Fig. 2:**
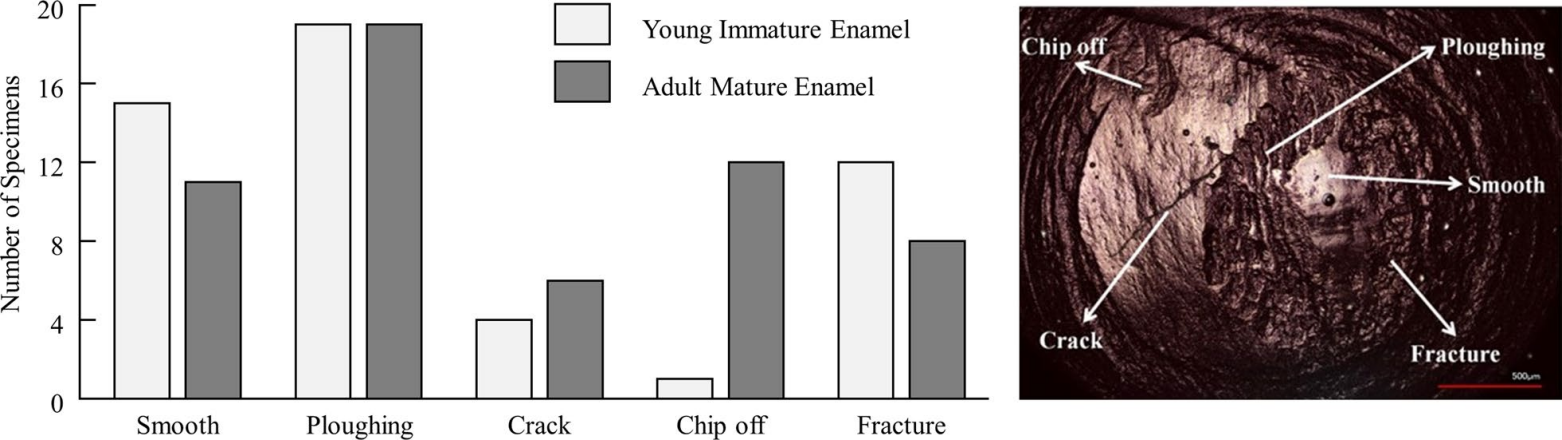
Wear characteristics of the upper specimens. Distribution of wear features in both groups

Four impressions were made from each upper specimen, at an interval of 5000 cycles (5000, 10,000, 15,000, and 20,000 cycles). After collecting the impressions, the samples were returned to the testing machine. A scanning laser confocal microscope (1LM 15 W, with Nikon lens, Nikon, Japan) was used to capture the impression of the wear area (minimum measurement scale, 0.302 µm). The wear characteristics, which included the ploughing, smoothening, cracking, chipping, and fractures, were examined, the change in the width and length of the wear areas was calculated (Fig. 2). Finally, few upper specimens were prepared and examined under a scanning electron microscope (SEM; H-4500, Hitachi High-Technologies Corporation, Tokyo, Japan) at magnifications of × 5000, × 10,000, and × 20,000 to examine the relationships between the microcracks and the enamel microstructures.

#### Lower specimens

The lower specimens were examined using a three-dimensional (3D)-colored laser microscope and a Profilometer (Keyence VK 200–300) at a magnification of × 20 and minimum scale accuracy of 0.001 µm on the Z-axis. The lengths and widths of the wear areas and craters were measured. Additionally, the maximum profile of the wear crater on the longitudinal section was determined. 3D image buildup of the wear surface was carried out, and the images were analyzed to evaluate the presence and dimensions of the craters and the fatigue cracks.

### Statistical analysis

SPSS software (IBM SPSS Statistics for Windows, version 24) was used for this study’s statistical analysis. Kolmogorov–Smirnov test and the Shapiro–Wilk test were used to test the normality of the data. The upper specimens’ wear areas, the lower specimens’ wear areas, crater widths, and crater depths were analyzed via multiple comparisons using the Mann–Whitney U test. The differences were considered statistically significant if the *p*-value was < 0.05.

## Results

### Upper specimens

#### Wear characteristics

The impression images revealed the type of wear that occurred in the specimens at the microscopic level. The types of wear were classified as ploughing (micro-cutting), smoothening (polishing of the surface), cracking, fracture (delamination or spalling), and chipping. The immature (IM) group’s specimens showed more resilience and resistance toward chipping; the enamel of only one specimen was chipped in the IM group compared to 12 in the mature (M) group. Ploughing and smoothening occurred in both groups (Fig. 2).

#### Wear areas

**Fig. 3:**
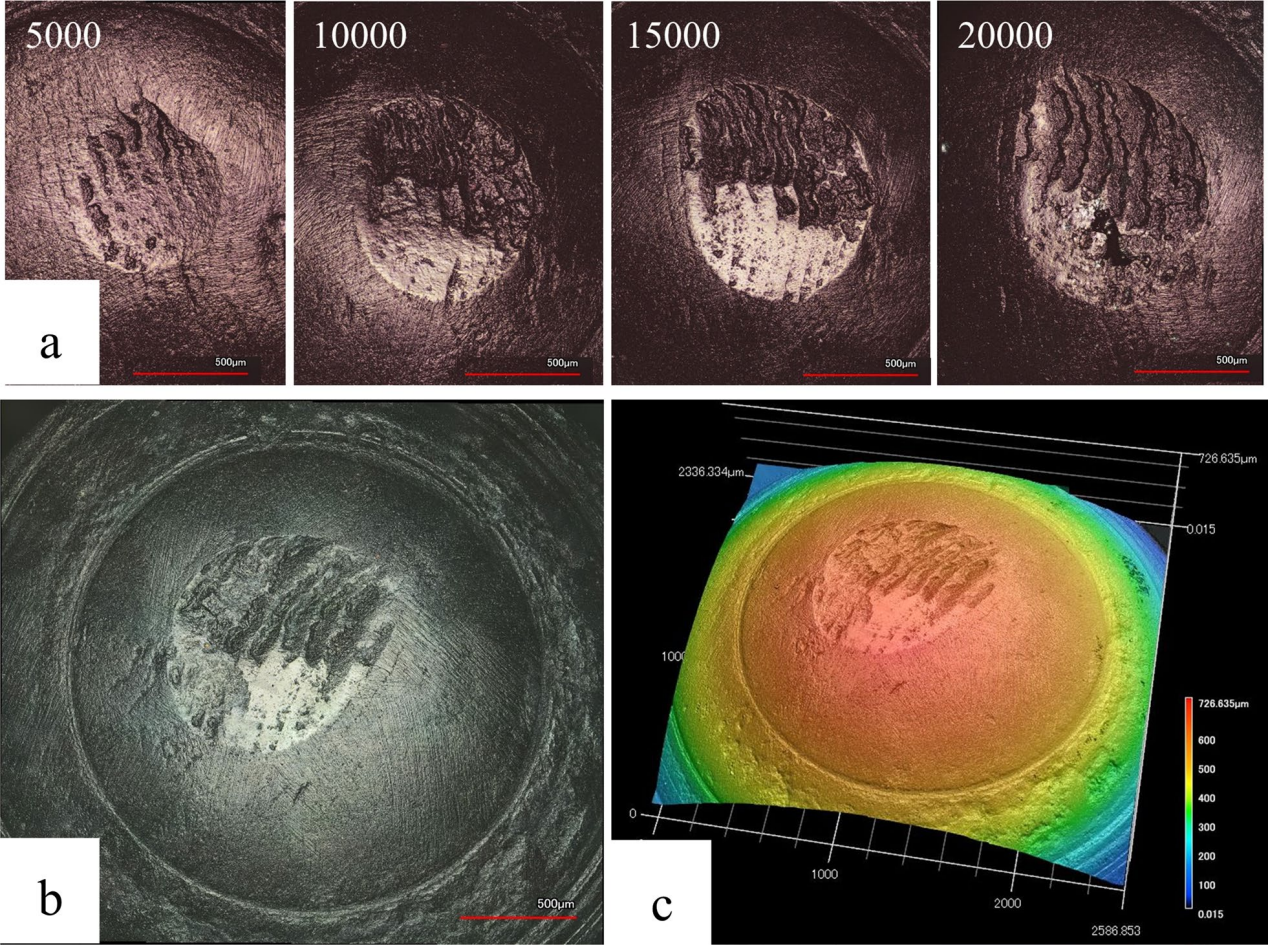
Microscopic imaging of an immature (IM) upper specimens; **a** laser confocal microscopic image of impressions of the upper IM specimen Scanned by laser confocal microscopy after every 5000 cycles of the impact sliding wear test; **b** after 20,000 cycles by a 3D-colored laser microscope; **c** 3D buildup image of the same upper IM specimen after 20,000 cycles

**Fig. 4:**
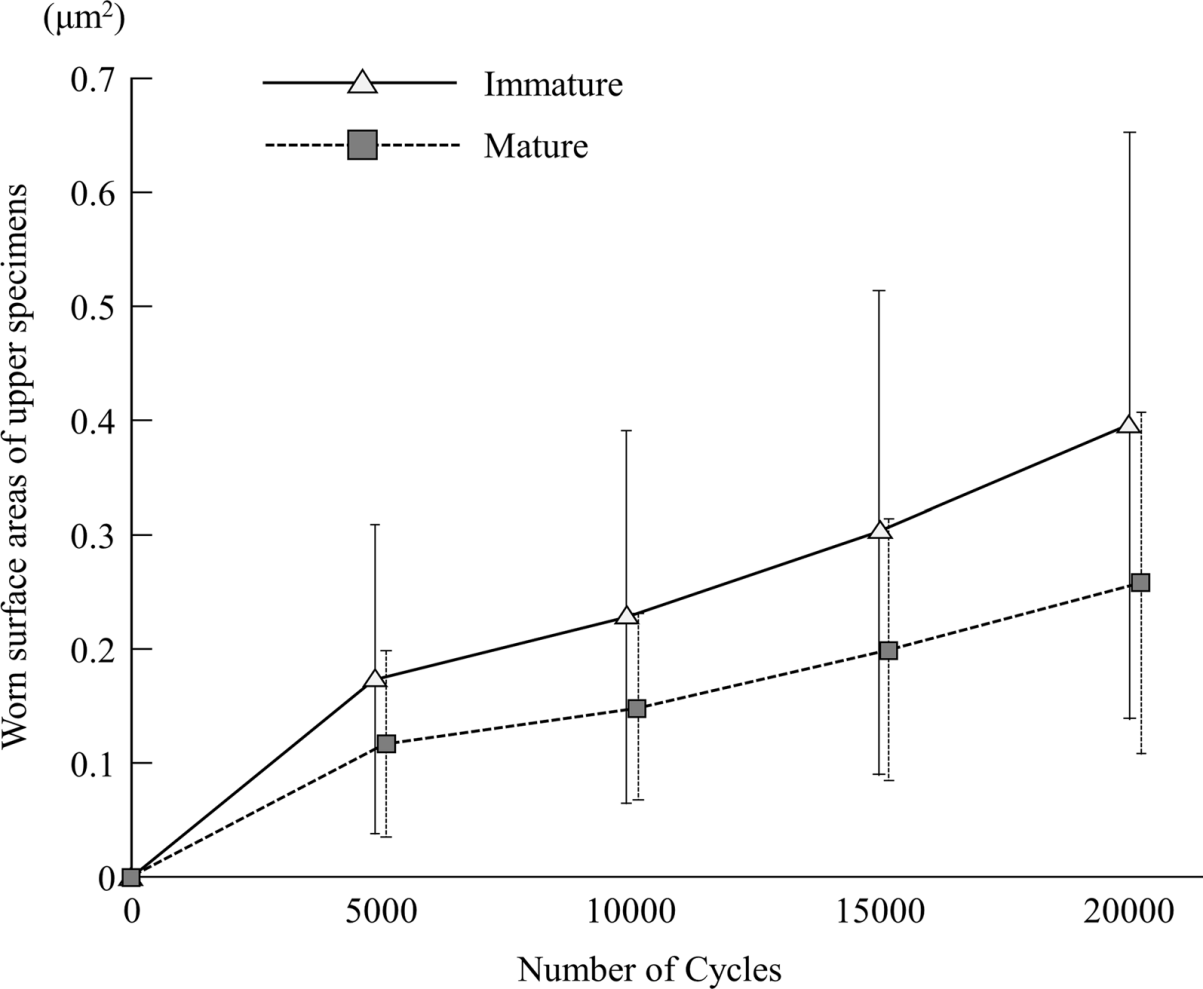
Analysis of the wear areas on the upper specimens after every 5000 cycles for the immature and the mature group

Four images were obtained from each upper specimen representing the wear after 5000, 10,000, 15,000, and 20,000 cycles (Fig. 3). The form of wear was clearly visible on the impression material and well magnified to show the exact size. The images were analyzed to calculate the incremental increase in the width and length of the wear areas (µm^2^) after every 5000 cycles in both groups (Fig. 4). No significant difference was observed between the IM and M groups (*p*-values were 0.393, 0.280, 0.280, and 0.247 for the 5000, 10,000, 15,000, and 20,000 cycles, respectively). The wear areas in the IM group demonstrated a slightly steeper increase in size after the first 5000 cycles than those in the M group.

### Lower specimens

#### Wear characteristics

**Fig. 5:**
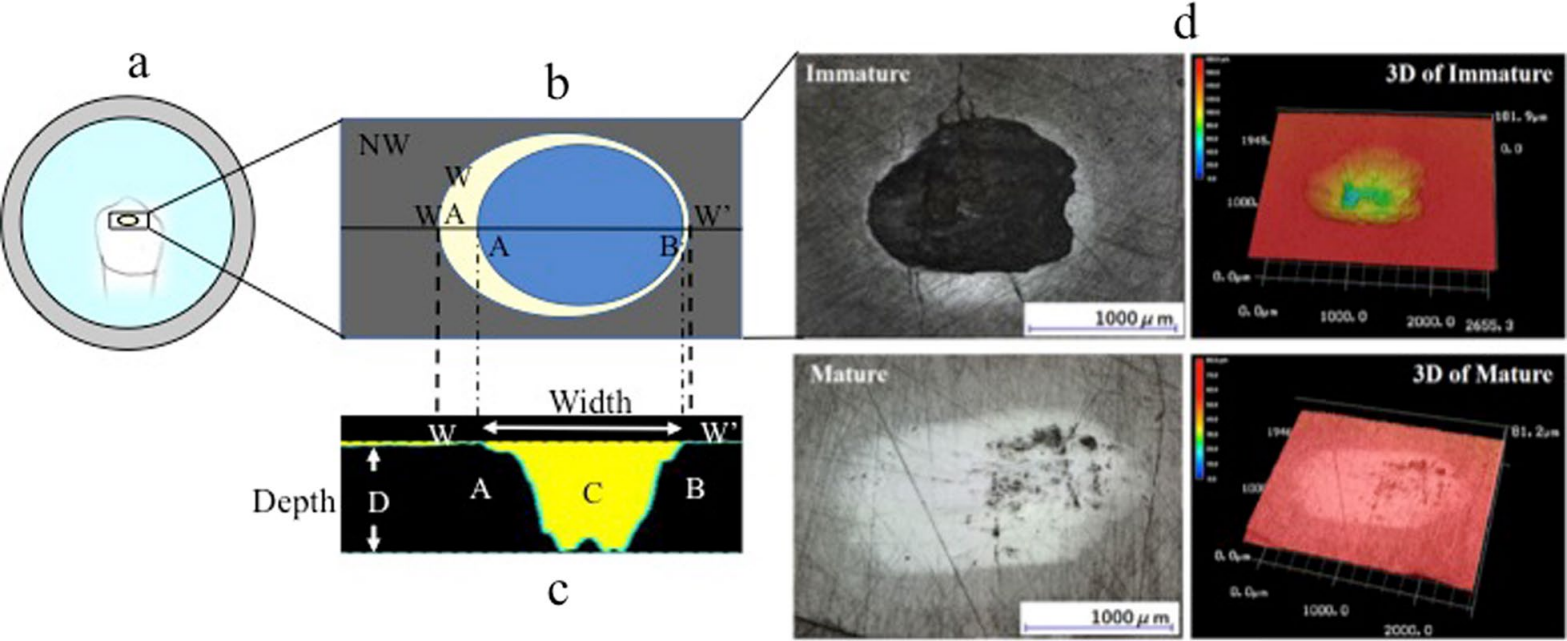
**a** Schematic illustration of the top view of the lower specimen after the impact sliding wear test; **b** classification of the findings in the insert area (NW: Non-worn enamel surface, WA: wear area, W and W’ indicate the long axis of the enamel wear area); **C** cross-sectional image of craters on the worn enamel surface obtained by color 3D laser scanning microscopy,(C: The distance between A and B was assumed to be the width of the crater, D: distance from the deepest point of the crater to the surface); **d:**Imaging of the lower immature (IM) and mature (M) specimens using a 3D colored laser microscope and a Profilometer

The IM group’s wear profile showed rough, deep, and irregular craters in all specimens. The M group had a flat and smooth wear profile without craters’ formation (Fig. 5).

#### Depth and width of craters

**Fig. 6:**
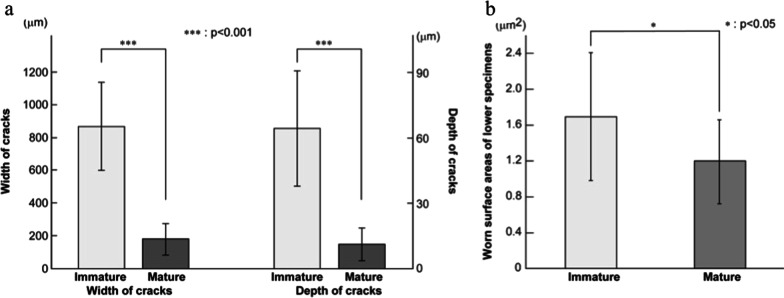
Analysis of wear features in the lower specimens; **A** the depth and width of craters/cracks in the mature (M) and the immature (IM) group; **B** analysis of the wear area in the lower specimens in IM and M groups

The mean values of the widths of each crater were 867.5 ± 537.7 μm and 181.9 ± 192.3 μm in the IM and M specimens, respectively (Fig. 6A). The mean values of the depths of each crater were 64.3 ± 52.8 μm and 11.2 ± 14.9 μm in the IM and M specimens, respectively. Significant differences in both width and depth were observed between the IM and M groups (*p* < 0.001).

#### Wear areas

Likewise, there was a significant difference in wear area between the IM and M groups. The craters were extremely wide and deep, occupying many wear areas in the IM group (*p* = 0.017) compared to the much shallower and narrower craters in the M group (Fig. 6B).

**Fig. 7:**
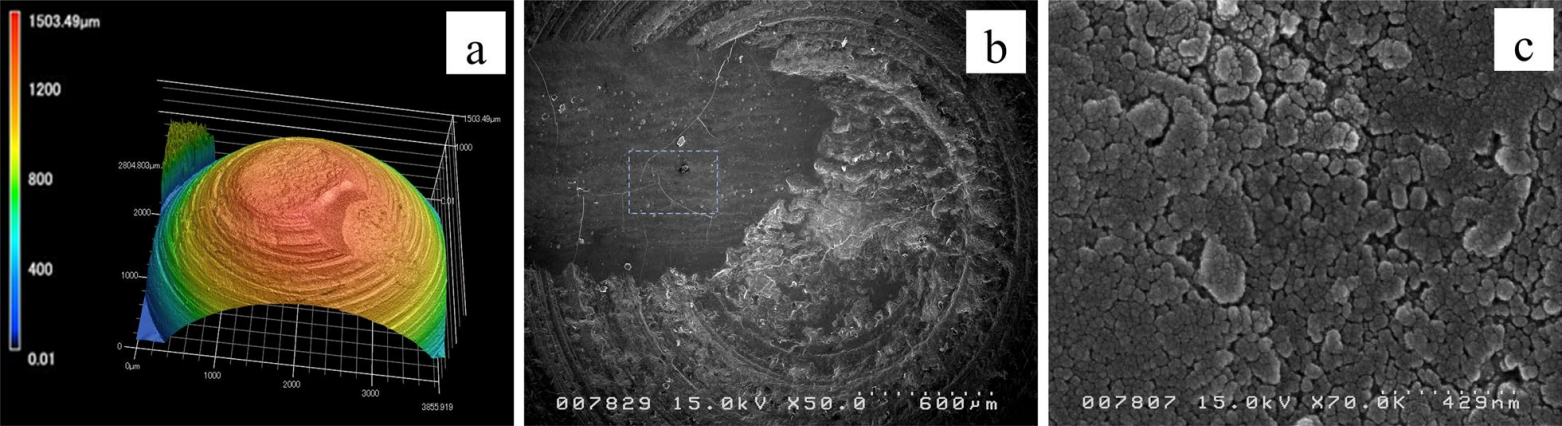
Relationship between the wear features and the enamel microstructures; **a** 3D image of the mature enamel from an upper specimen showing chipping; **b** SEM image of the same specimen showing microcracks on the enamel surface beneath the chip; **c** The enamel crystals are intact with signs of compression and detachment due to the applied forces

SEM imaging (Fig. 7a–c) revealed that the enamel crystals were detached at the microscopic level but intact in the M group; the crystals were highly packed with limited inter-crystallite spaces (Fig. 7C). The IM group had a higher number of microcracks, and the enamel crystals suffered more damage due to the crushing forces when compared to the M group. The inter-crystallite spaces were larger than those in the M group.

## Discussion

Many studies have investigated tooth wear in mature teeth [4, 5], however scientific literature of tooth wear in immature teeth in children and adolescents is scant [6]. In this study, the wear characteristics of immature enamel to these of mature enamel were compared, by subjecting enamel specimens derived from different age groups (immature open-apex premolars, mature closed-apex premolars) to cycles of simulated impact and sliding wear testing under controlled conditions Our findings confirmed that the increase in the enamel’s post-eruption age affected its wear behavior and, subsequently, its wear characteristics.

In this study, the mature group specimens were obtained from premolars belonging to patients aged 25–30 years. In a previous study, which compared the wear behavior of permanent teeth at different ages (18, 35, and 65 years) against titanium alloy, the mature premolars were obtained from different age groups (18–35 years) [8]. Although permanent teeth at the young (18 years) and middle (35 years) ages were reported to have better wear-resistance than old ages, immature teeth were not included [8]. The immature enamel specimens in this study were obtained from premolars extracted from orthodontic patients aged 9–15 years. Our findings indicate permanent immature teeth are less wear resistant than mature permanent teeth, thus a hierarchy of wear resistance in permanent dentition might exist as immature, young mature, middle-age mature, and old-age mature dentitions.

Ploughing was the main wear feature in the upper specimens; where most of specimens demonstrated ploughing (Fig. 2). This was consistent with Zheng and Zhou’s findings [8] but in contrary with another study that found delamination and fatigue wear were more prominent [15]. Our results showed that the mature group’s enamel showed smaller wear areas, but the upper specimens’ hemispheric shapes resulted in more chipping in the oblique direction from the tip (Fig. 6). The upper specimen’s hemispheric shape imitated the shape of a tooth cusp and was created with the same custom-made standardized bur. Therefore, the difference in chipping between the immature and mature groups was not related to the experimental procedures used as all specimens had the same shape and size. In a previous review [1], chipping was considered a “mesofracture.” Mesofracture starts on the indented enamel surface close to the edge of cusp. However, fractures are directed from the inside of the enamel toward a closer axial surface removing small pieces of enamel, but this does not continue to cause division of the entire crown or loss of large enamel slabs [1]. In this study, chippings were more observed in the upper mature specimens. This will prevent the continuation of the fracture to cause a total crown split or loss of larger enamel slabs.

We obtained impressions of each upper specimen after every 5000 cycles. Unlike previous quantitative studies, microscopic images used for quantifying wear measurements were obtained directly from the impressions rather than from epoxy replicas [4, 16]. The impressions were examined immediately after they had been produced to reduce any dimensional deformation. The forms of wear and cracks were clearly represented in the impression material. This direct observation of the silicon impression material proved to be useful in our analysis of wear features. In a previous study, it has been proved that Silicone based compound as a surface replication material was able to consistently detect cracks as small as 0.005 inches (125 μm) long and pit-like surface defects as small as 0.001 inches (25 μm) long with better handling properties compared to other materials [17].

The lower specimens’ wear features in the immature group comprised rough, deep, and irregular craters/cracks with multiple microcracks. On the other hand, the M group had smooth, shiny, and shallow wear areas without craters formations. The differences in the abrasion patterns in the two groups might be attributable to differences in the enamel’s physical properties or the effect of posteruptive age [6].

Palti et al. [18] revealed differences in the superficial microhardness of specimens among different eruptive ages (before eruption in the oral cavity, 2–3 years after the eruption, 4–10 years after the eruption, and more than 10 years after the eruption); their results indicated an increasing mineralization behavior of the enamel. The porosity [19, 20] and inter-crystallite spaces [21] of the enamel were large during an eruption but decreased with the increase in the teeth posteruptive ages. These characteristics are directly related to the hardness of the enamel due to posteruptive maturation. Furthermore, there is a direct relationship between the mineral volume and the hardness of the enamel [22, 23]. These factors are related to the exposure of the enamel to the normal dynamic processes and the oral-salivary environment [21, 24]. The difference in wear characteristics between the specimens in the mature and immature groups, as seen in the images, was presumed to be due to the influence of these physical properties. In this study, the inter-crystallite spaces in the immature group’s enamel were larger than those in the mature group, and the cracks occurred along these inter-crystallite spaces.

Lucas and van Casteren’s study explains the process, mechanism, and classification of enamel wear and microcracks on the enamel surface [1]. They stated that microcracks occurred when small hard particles contacted the enamel’s surface; cuts are made between the enamel rods when these particles slide on the enamel surface. Borrero-Lopez et al. referred to the increased microcrack development and coalescence process as a microcracking mode and/or severe wear [25]. Ijbara et al. reported that immature teeth are prone to subsurface cracking when compared to mature teeth [6]. The present study’s findings suggested that the microcracks on the surface overlapped with those on the subsurface, leading to load fatigue, peeling off of the enamel, and large craters. The difference in the wear appearance in the immature and mature groups may be due to differences in the chemical and physical properties of the enamel related to its posteruptive maturation.

Wear involves a 3D loss of substance; therefore, the volume is the preferred parameter for quantifying wear. Delong stated that 3D scanning is preferred for measuring wear because it provides quantitative, accurate, and storable data [26]. In the current study, a scanning, colored nano-microscope with a Profilometer was used. The microscope allowed intra-examiner calibration due to its software objectivity. Furthermore, 3D constructed images confirmed the depths and widths of the craters. Also*,* the types of wear could be classified in more detail as follows: ploughing (micro-cutting), smoothening (polishing of the surface), cracking, fracture (delamination or spalling), and chipping.

Dahl et al. [2] discussed that the oral function is considered a three-body phenomenon, i.e., oral wear includes two surfaces of the teeth with a food bolus. In this study, the food bolus was represented by the PMMA slurry (particles of 50 µm diameter) between the specimens during the test. The presence of both the slurry and the immersion in water with controlled temperature during the test mimicked the oral conditions. A recent study discussed the biological effects of consumed substances by teenagers [27]. Not only different materials have different abrasiveness which can alter wear rate, they have harmful chemical properties that can cause inflammation to the oral cavity tissues with apoptosis of vital tissues [27]. Regarding the load and number of cycles selection, Leinfelder and Suzuki [14] demonstrated that the 400,000 cycles and 75 N loading used for the permanent dentition correlated with the in vivo values gathered over a 3-year period. We selected experimental parameters of 30 N to simulate the conditions in the oral cavity of a child/young adult. Therefore, 20,000 cycles and 30 N were suggested to exhibit features of early wear [4, 6].

The factors that influence wear include age, sex, ethnicity, diet, location, type of occlusion, parafunctional habits, materials, and even the antagonist tooth’s shape and posteruptive age [3, 4, 27]. The causes of loss of dental hard tissues are multifactorial and co-existing [28]. Accordingly, restoring dental caries, trauma, and defects in immature teeth in the clinical practice, the repair material should be chosen carefully to avoid inappropriate wear of the opposing natural immature teeth. The use of suitable restorative materials for immature teeth should be investigated in the future as the results of this study showed a difference in the wear characteristics according to the posteruptive age which refuted our null hypothesis.

### Limitations

The authors acknowledge the observational, extrinsic approach of this study and the need of further investigations. Further three-dimensional imaging, measurements and analysis of the upper specimens were faced with difficulties in reference plane standardization, due to the dome shape preparations. Further comparisons in wear factors such as load, temperature, and two-body vs three-body tests are essential in understanding differences in wear behavior in different age groups. Our future research plans include micromechanical and chemical analytic approaches in examining the wear characteristics in relation to posteruptive age. Tests as nanoindentation, microtoughness, and microfracture on sound, artificial caries and carious tooth surfaces might be included. These approaches would reveal differences in the intrinsic toughening mechanism of enamel, that is, the exact role of the enamel surface and subsurface microstructures in wear behavior in relation to the posteruptive age.

## Conclusion

Within the limitations of this study, the following conclusions were reached: The immature enamel had different wear characteristics compared to mature enamel. The immature enamel demonstrated wider and deeper craters; the lower specimens’ wear areas were significantly larger than those in mature enamel. These findings indicated that immature teeth should be repaired using restorative materials with mechanical properties comparable to those of the immature enamel.

## Data Availability

All collected data analyzed during this study are included in this published article. Some datasets are available from the corresponding author on reasonable request.

## Abbreviations

°C: Degree celsius
IM: Immature
ISWT: Impact sliding wear testing
M: Mature
SEM: Scanning electron microscope
SPSS: Statistical package for the social sciences
3D: Three-dimensional
